# Associations across food consumption, diabetes prevalence and the environmental impacts: Evidence from China

**DOI:** 10.1371/journal.pone.0334136

**Published:** 2025-10-14

**Authors:** Bingtao Su, Xianqiang Cao

**Affiliations:** 1 School of Philosophy and Social Development, Shandong University, Jinan, China; 2 Shenzhen Research Institute, Shandong University, Nanshan, Shenzhen, China; 3 School of Political Science and Public Administration, Shandong University, Jimo, Qingdao, China; Center for Research and Technology Transfer, VIET NAM

## Abstract

Dietary choices are shifting globally in ways that are directly affecting both the environment and human health. Building on the Diabetes Epidemiological Survey, and the latest data from the China Statistical Yearbook, this study quantified Chinese people’s dietary ecological footprint per capita (EFP) and the greenhouse gas (GHG) emissions at the provincial level with different prevalence of diabetes and prediabetes. Results demonstrated that groups with a lower prevalence of diabetes showed lower dietary EFP, while the dietary EFP and GHG emissions peaked among the prediabetes group with a prevalence of 32.7%−34.9%, and then decreased significantly with the increasing prevalence of prediabetes. The increasing prevalence of diabetes was tightly linked to increased consumption of pork, poultry, seafood, eggs, and vegetables. Moreover, the over-consumption of meat, oil, and cereal, together with the under-consumption of vegetables, fruits, eggs, and milk, will further increase both the prevalence of diabetes and environmental degradation. Yet, people’s concern for their health will drive them to pursue a healthier diet, which in turn will promote environmental sustainability. Findings in this study can help to reshape the dietary patterns that can effectively reduce the environmental and health impacts not only in China but also in other countries with accelerated diet-environment-health trilemma.

## Introduction

Food consumption links human and environmental health [1–4]. Dietary shifts from plant-based to animal-based food increase environmental impacts, while excessive red meat intake also raises health concerns [5,6]. Over the last 60 years, animal-based product consumption has surged worldwide [7]. This directly resulted in the dietary-related risks ranking top among all the health risks including obesity, heart disease, and diabetes [6]. Diabetes is a serious public disease that is highly related to modern nutritional habits and dietary choices [8,9]. According to IDF (International Diabetes Federation) Diabetes Atlas 10^th^ edition 2021, an estimated 537 million people have diabetes, and over 6.7 million people aged 20–79 died from diabetes-related causes in 2021 [10]. China is the country with the largest number of adults with diabetes aged 20–79 years in 2021. There were 140.9 million Chinese people with diabetes in 2021, and this number will become 174.4 million in 2045. Simultaneously, China is also the country with the highest number of people (72.8 million) with undiagnosed diabetes in 2021 [10]. Due to its large population, China has the highest annual number of deaths from diabetes, at approximately 1.4 million [10].

Previous research has demonstrated significant positive correlations between diabetes and red meat, fish, and white rice intake [11,12], and significantly negative correlations between diabetes risk and the consumption of dairy products, vegetables, and fruits [9,13–15]. The rising incomes and urbanization in China are driving a national dietary transition in which plant-based products (e.g., cereals) are replaced by pork, poultry, and seafood [16], and the consumption of per capita meat consumption in China is strongly increasing [17]. Evidence has shown that China has entered an era dominated by animal-based products [3]. The over-consumption of cereal and meat, and the deficiencies of dairy, vegetables, and fruit may increase the risk of diabetes in contemporary China [18]. Given that dietary intake is a major modifier in diabetes management [9], the explorations of sustainable and healthy dietary choices are of vital importance to controlling the increasing number of diabetes in China.

Besides human health, the way of food consumption is increasingly affecting the environment [16]. Previous research has estimated that greenhouse gas (GHG) emissions from food systems account for 35% of global total anthropogenic GHG emissions [19]. The largest number of people and the over-consumption of meat in China has contributed to environmental degradation and even global warming [3]. For instance, China has expanded its per capita pork consumption by two-fold and beef consumption by five-fold since 1971 (Westcott, 2014). The GHG emissions of food consumption for an urban Chinese individual rose by 10.0 g CO_2_e per year, and for a rural individual rose by 18.0 g CO_2_e per year between 1997–2011 [16]. Additionally, China has witnessed the largest scale and most rapid urbanization during the last several decades [20]. Urban food consumption, with higher energy density and more animal foods, has reshaped people’s dietary patterns [16], which will undoubtedly increase the environmental burden and health risks [21]. Given that the current dietary choices directly link and negatively affect human and environmental health, the national dietary transition is a great challenge facing humanity, especially in countries like China [2].

Recently, transformation to healthy and environmentally friendly diets has been encouraged at the national scale for a more sustainable future. Here, we investigated the health-environment-nutrition relationships in response to food consumption by Chinese people. Based on the Diabetes Epidemiological (DE) survey (2015–2017) and the prevalence of diabetes and prediabetes [22], we divided the 31 provinces of mainland China into five groups, respectively. Using data from the China Statistical Yearbook (CSY), we estimated Chinese people’s per capita food consumption and compared the different dietary ecological footprints per capita (EFP) and GHG emissions per capita across five groups of diabetics and prediabetics. We further investigated their dietary patterns based on the consumption quantity of each food item and evaluated the deviation of people’s food intake by comparing their actual food consumption with the recommended standard from the 2022 Chinese Dietary Guidelines (CDG). To determine food consumption behaviors and simultaneously guarantee environmental and human health, we also explored the deviation of diabetes and prediabetes groups’ EFP and GHG emissions from the recommended nutritional requirement for both the general public (CDG) and the diabetics (Chinese Diabetes Society, CDS). We finally analyzed the triangle relationship among different food items consumption, diabetes and prediabetes rates, and dietary EFP/GHG emissions. The results can help to reshape the dietary patterns that effectively reduce the environmental and health impacts not only in China but also in other countries with accelerated diet-environment-health trilemma.

## Materials and methods

### Ethics statement

This study was conducted in compliance with the relevant guidelines established by the Ethical Review Committee of Shandong University.

### Study areas

To observe the relationship between dietary environmental impacts (i.e., EFP and GHG emissions) and different prevalences of diabetes and prediabetes, we divided the 31 provinces of mainland China into five groups (i.e., groups with the lowest, low, medium, high, and highest prevalence of diabetes; groups with the lowest, low, medium, high, and highest prevalence of prediabetes), respectively. This partition was identified based on the DE survey, which included 31 provinces of mainland China with a total of 75880 people based on the gender and age composition of urban-rural ratio and each community [22]. According to the DE survey, the prevalence of the five diabetes groups was 6.2%−10.4% (six provinces), 10.6%−11.9% (six provinces), 12.0%−12.9% (six provinces), 13.4%−14.4% (six provinces), and 14.4%−19.9% (seven provinces); while the prevalence of the five prediabetes groups was 17.2%−29.8% (six provinces), 29.9%−32.0% (six provinces), 32.7%−34.9% (six provinces), 34.9%−39.8% (six provinces), and 40.1%−54.5% (seven provinces).

### Data sources

To be consistent with the period of the DE survey, and simultaneously ensure the accuracy of the comparative data of the environmental impacts and the prevalence of diabetes and prediabetes, the food consumption data of this study was from 2015 to 2017. To quantify the per capita dietary environmental footprint, we used the individual-level data of the food intake from the China Statistical Yearbook (data in S1 text). Seven animal-based (pork, beef, mutton, poultry, seafood, egg, and milk) and five plant-based (cereal, vegetables, fruit, oil, and sugar) food items were introduced in this study. Further information from the Global Footprint Network, the Food and Agriculture Organization Statistics (FAOSTAT), the database of Our World in Data, and published papers [3,23–27] was used to calculate the dietary EFP and GHG emissions of food consumption in China. Based on the data of 2022 CDG and CDS, we also explored the deviation of individual consumption of each food item by comparing the actual intake with the recommended intake for the general public and diabetics.

### The calculation of the dietary EFP and GHG emissions

The ecological footprint (EF) and GHG emissions are good measurements of environmental degradation since they both embody the detrimental impacts of economic activities on nature [19]. The EF measures human use of nature in six main ecologically productive land types: cropland, grazing land, forest land, fishing areas, built-up land, and energy land [28]. According to the food product items (cereals, oils, vegetables, fruit, pork, beef, Mutton, poultry, milk, egg, and seafood) that we introduced in this study, cropland, grazing land, and fishing land were used to calculate the dietary EFP. GHG emission is the amount of carbon dioxide emitted due to human activities (e.g., food consumption) [29]. The analysis of the GHG emissions of food consumption through life cycle assessment (LCA) has been rapidly raising interest during the last decade [30,31]. The LCA is a method that addresses the environmental impacts of a product or a service, and it was used in this study to quantify the GHG emissions regarding diabetes and prediabetes groups’ food consumption. The GHG emissions from the process of food production were also included in the calculation.

The calculation of the dietary EFP is shown below following our previously established method [3]:


$$EFP=\sum \frac{{\text{C}}_{i}}{\text{Y}i}r_{j}$$ (1)

Where i is the number of food consumption items (i=1 to 12); $C_{i}$ is per capita consumption of food item i  (kg, i=1 to 12); $Y_{i}$ is the yield factor for the land type of food item i  (kg/ha), and $r_{j}$ is the equivalence factor of land type j (j=1 to 3).

The yield factor is the ratio of national to global average yields [32]. The equivalence factor is the ratio of the average productive capacity of an area and the whole world [33].

The GHG emission can be calculated by the equation below [34,35]:


$$\text{GHG}=\sum C_{i}\times EI_{i}\;$$ (2)

Where i is food consumption items, $C_{i}$ is per capita consumption of food item i (kg, i=1 to 12), ${EI}_{i}$ is the GHG emission intensity for food item i (kg CO_2_/kg).

### Statistical analysis

To detect the different environmental impacts of diabetes and prediabetes groups, a series of statistical analyses were conducted. The average dietary EFP and GHG emissions, and the 95% confidence interval of each diabetes and prediabetes group were calculated, and separate one-way ANOVAs were used to explore the extent of the differences of the dietary EFP, GHG emissions, and the quantity of food consumption across different diabetes and prediabetes groups, respectively. Pearson correlation analysis was used to measure the degree of association across the prevalence of diabetes and prediabetes, the dietary EFP and GHG emissions, the consumption quantity of each food item, and other factors (i.e., economic and geographical indicators). Fisher’s procedures were performed to reduce type-I errors. The data in this study followed a normal distribution or could be converted to a normal distribution, and the Levene test showed heterogeneity of variance. Data were analyzed with SPSS 24.0 statistical software and Origin 8.0 mapping software. All results were based on two-sided tests, and the value of p < 0.05 was considered significant.

## Results

### The dietary EFP and GHG emissions of diabetes and prediabetes groups

**Fig 1:**
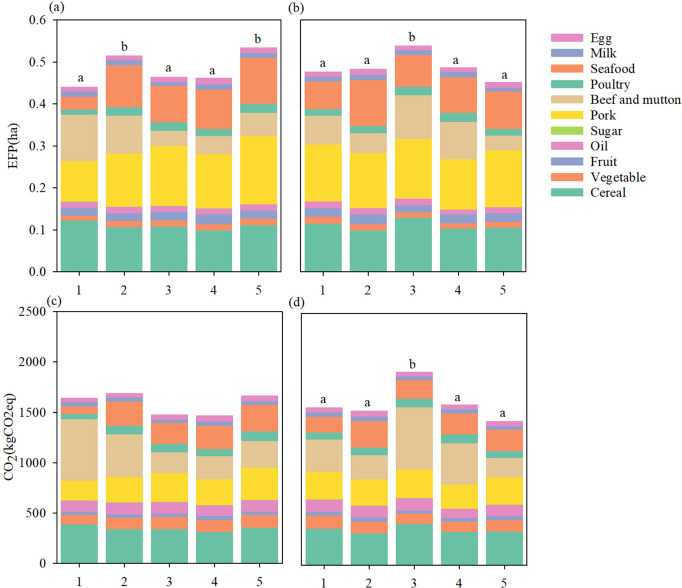
The environmental footprint of diabetes and prediabetes groups.

The dietary EFP and GHG emissions were quantified for five groups of individuals with diabetes and prediabetes in China, and results showed a lower EFP by the group of LSD and lower EFP and GHG emissions by LSPD and LPD groups. Results also demonstrated significant differences of the total and animal-based EFP across five diabetes groups, yet no significant difference regarding plant-based EFP was found. Specifically, LD and HSD groups had higher EFPs (0.514 ha and 0.533 ha, respectively), which is mainly driven by animal-based EFPs (0.358 ha and 0.371 ha, respectively), than LSD, MD, and HD groups. For a broader picture, compared to the lower prevalence of diabetes groups, the higher groups had relatively higher EFP scores for pork, poultry, and seafood (all p < 0.05). However, we did not find any significant differences regarding their dietary GHG emissions. Concerning the prediabetes groups, the MPD group showed higher scores of total (0.554 ha) and animal-based EFP (0.383 ha) and total (1910 kg CO2eq) and animal-based GHG emissions (1251 kg CO2eq) than the other four groups, but we did not find any significant differences of the EFP of pork (p = 0.388), poultry (p = 0.126) and seafood (p = 0.070) across five groups. LSPD and MPD groups showed higher scores of plant-based EFP and GHG emissions than the other three groups. The main sources of the dietary EFP and GHG emissions of all diabetes and prediabetes groups were pork, cereal, seafood, beef, and mutton; while EFP and GHG emissions from vegetables, fruit, egg, and milk were very small. Overall, both EFP and GHG emissions from animal-based products have exceeded those from plant-based products for all ten groups (Fig 1).

### The total, animal and plant-based product consumption of diabetes and prediabetes groups

**Fig 2:**
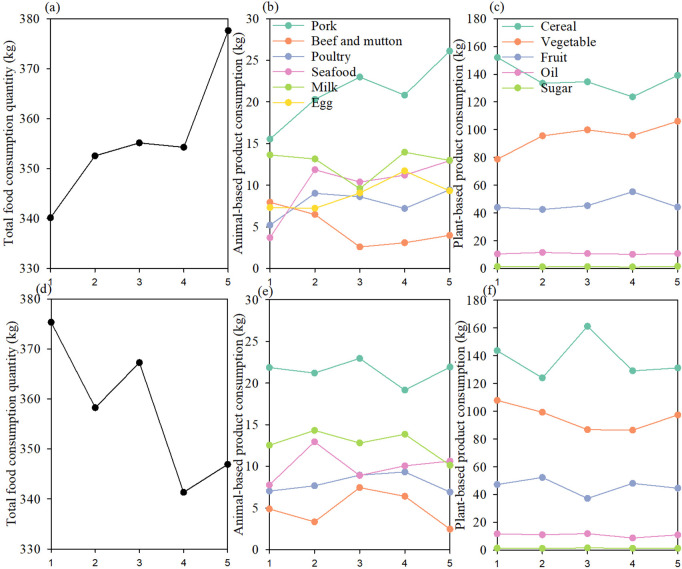
The food consumption quantity.

With the increasing prevalence of diabetes, people’s total food consumption quantity increased significantly (p < 0.001). Specifically, with the increasing prevalence of diabetes, people’s consumption of pork, poultry, seafood, and vegetables also increased, and the growth rate of pork is faster than the other food items. Conversely, with the increasing prevalence of prediabetes, people’s food consumption quantity decreased significantly (p < 0.001). Specifically, the LSPD and LPD groups showed higher consumption of vegetables, the MPD group showed higher consumption of cereal, beef, and fruit, while the HPD group showed lower consumption of oil and higher consumption of mutton, (all p < 0.05) (Fig 2).

### The deviation of food consumption by diabetes and prediabetes groups

**Fig 3:**
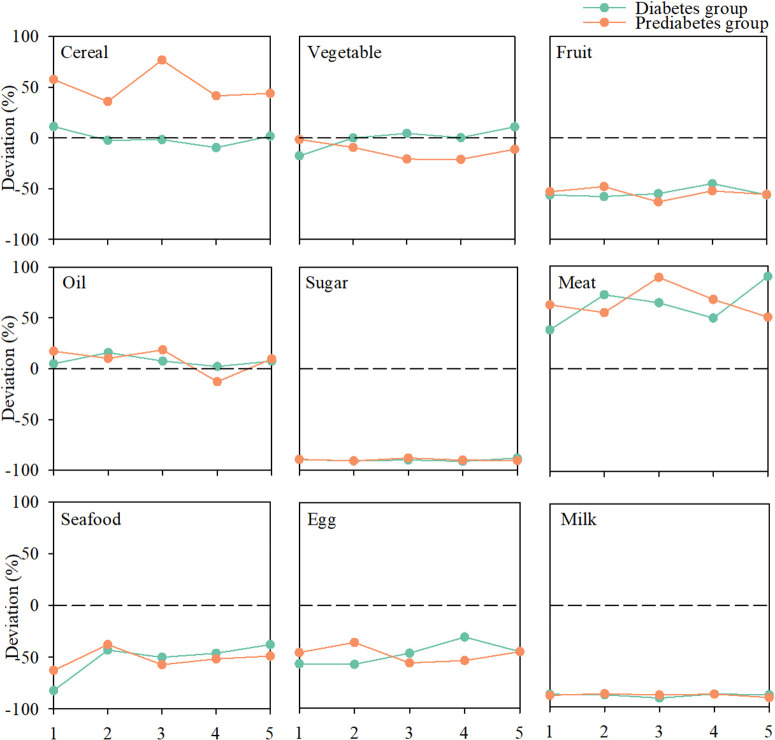
Deviation from the balanced dietary patterns outlined in the 2022 CDG.

In this study, we assessed deviations in food intake by comparing the consumption patterns of individuals with diabetes and prediabetes to the recommendations outlined in the 2022 CDG (Fig 3). Results showed that the present food consumption structure in China is widely divergent from the standard proposed by CDG. For example, the overconsumption of meat, oil, and cereals—combined with the insufficient, albeit increasing, intake of vegetables, fruits, eggs, and milk—is likely to further exacerbate both the prevalence of diabetes and environmental degradation.

Meat is the most excessively consumed food item and based on the daily requirement, diabetes and prediabetes groups, on average, consumed 62.56% and 64.59% more than they needed. More surprisingly, the over-consumption of meat for MPD groups reached 88.79%, and for HSD groups even reached 89.74%. The over-consumption of cereal is also serious for prediabetes. For example, the MPD group consumed 76.66% more than they needed. Yet, the consumption of cereal by diabetes groups is moving closer to the recommended diet pattern over the years. Overall, almost all groups experienced underconsumption of fruit, sugar, seafood, egg, and milk. Specifically, the underconsumption of milk and sugar was stable and severe for all diabetes and prediabetes groups, consuming 86% to 92% less than they needed. Diabetes and prediabetes groups showed a similar trend to underconsumption of fruit and eggs. Furthermore, the diabetes groups showed a quick increase in vegetable consumption, and a slow but steady increase in fruit, seafood, and egg consumption (Fig 3).

### The correlation analysis

**Fig 4:**
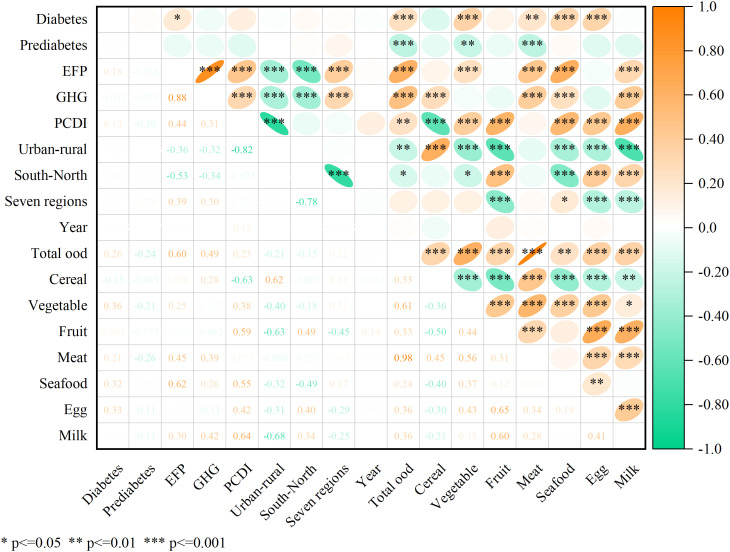
The correlogram.

Correlations were examined among diabetes and prediabetes prevalence, dietary EFP and GHG emissions, overall and specific food consumption (seven food items), as well as various economic (PCDI and urban/rural status), geographical (southern/northern divisions and seven regions), and temporal (years) factors (Fig 4). With the increasing prevalence of diabetes, people’s dietary EFP also increased significantly. Results also showed significant correlations between food consumption and the prevalence of diabetes and prediabetes, as well as between food consumption and the dietary EFP and GHG emissions. Specifically, the increasing prevalence of diabetes is strongly associated with higher consumption of total food, including pork, poultry, seafood, eggs, and vegetables, whereas the rising incidence of prediabetes is closely linked to lower intake of total food, particularly oil and vegetables.

The dietary EFP was positiviely correlated with total food consumption and the consumption of vegetables, meat, seafood, and milk, and the dietary GHG emissions were positively correlated with the total food consumption and the consumption of cereal, meat, seafood, and milk (all p < 0.001). The economic and geographical indicators significantly correlated with the dietary EFP and GHG emissions, yet we did not find any significant correlations between economic and geographical indicators and the prevalence of diabetes and prediabetes (Fig 4).

## Discussion

The increase in diabetes rates has occurred in parallel with changes in global food systems, which have driven higher consumption of animal-based and ultra-processed foods, resulting in a stronger environmental degradation [19,36]. Previous studies have found a significant correlation between food consumption and dietary environmental impacts [24,37], as well as the correlation between food consumption and diabetes rates [38,39]. Yet, information on the diet-environment-health trilemma is still limited. This study explored the significant pairwise correlations across food consumption, dietary environmental impacts, and the prevalence of diabetes and prediabetes in China. Results demonstrated that the diets of individuals with higher rates of diabetes cause larger EFP because of greater animal source products (i.e., meat, seafood, and egg). The over-consumption of meat and seafood in contemporary China has further contributed to the increasing prevalence of diabetes and environmental degradation. Although the dietary patterns in China are drastically changing and stunted development and undernourishment have been declining [16], the problems of unbalanced dietary structure, due to its attributes of environmental and human health, are still pressing and need to be addressed. This study illustrated the importance of considering the dietary pattern when studying its environmental and health effects, as associations with incident diabetes and EFP/GHG emissions.

Over the past decade, scientific interests and public awareness of the relationship between food consumption and human health, as well as food consumption and the environment have risen substantially [16,38,40,41]. Our results provide an independent confirmation of the association between dietary environmental impacts and incident diabetes. Findings demonstrated that the dietary EFP and GHG emissions changed with the prevalence of diabetes and prediabetes. For example, the LSD group showed lower consumption of total food and animal-based food products (e.g., pork, poultry, seafood, and egg), resulting in a lower dietary EFP than that of the HSD group. This finding suggests that the dietary patterns of the LSD groups are crucial for achieving a win-win situation for both environmental and health goals. The dietary EFP and GHG emissions peaked among the MPD group (i.e., prediabetics with a prevalence of 32.7% to 34.9%), and then decreased significantly with the increased prevalence of prediabetes. This inverted V-shaped curve indicated that the increasing prevalence of prediabetes was not always accompanied by the increasing environmental impacts. Conversely, when the prevalence of prediabetes increases to a certain level (ca. 35%), the dietary environmental footprint may decrease. Since the inherent synergies and trade-offs between reducing the dietary environmental impacts and the prevalence of prediabetes, ignoring such an environment-health nexus may lead to adverse effects [16]. Therefore, the dietary choices of the LSPD and LPD groups (i.e., provinces with the lowest and lower prevalence of prediabetes, and simultaneously with less environmental impacts) deserve to be explored to reduce environmental degradation and the risk of prediabetes. Our results confirmed LSPD and LPD gourps’ lower dietary environmental impacts due to their lower consumption of cereal and animal-based products (e.g., beef, poultry, and seafood) and higher consumption of plant-based products (i.e., vegetables and fruit). Therefore, a shift towards healthier diets could result in a considerable reduction of dietary EFP and the risk of diabetes.

Reducing the over-consumption of meat is beneficial for both the environment and human health in contemporary China. However, the dietary environmental impacts from animal-based products have exceeded those of plant-based products, confirming that China has entered an era dominated by animal-based food, or more specifically, pork-based food [3]. Pork is central to all discussions of food in the Chinese world, and the shortages of pork may spark street disorders and even threaten the stability of the state [3,42]. Pork consumption accounts for more than 60% of the total meat consumption in China [43]. These may verify the dominant role of pork regarding Chinese people’s dietary environmental impacts, and simultaneously explain the phenomenon of the over-consumption of meat. Compared with pork, Chinese people were less likely to consume poultry, and our findings showed that poultry consumption only accounted for one-fifth of the total meat consumption. A previous study has demonstrated a significant association between red meat intake and diabetes, and the non-significant association between poultry intake and diabetes [11]. Therefore, attempts to resolve the dilemma of the environment and health by replacing some pork consumption with poultry meat is important for Chinese people, and our findings verified this possibility. Previous research has revealed that consuming more white rice was positively correlated with the risk of type 2 diabetes mellitus, especially in Asian countries [12]. Our findings revealed that with the increasing prevalence of diabetes, people’s consumption of cereal decreased, while the consumption of pork increased dramatically, although pork was also demonstrated as a stimulating factor for diabetes. This finding demonstrated that Chinese people recognized the relationship between staple food and the diabetes risks, suggesting that people’s concern for their health may influence their dietary choices, resulting in the changes of the dietary environmental impacts. It also illustrated that more targeted dietary guidance (e.g., pork intake based on energy requirement and blood sugar level) should be formulated and popularized for diabetes and prediabetes people in China.

Due to the cooking methods, Chinese people were likely to consume more oil than the recommended standard. The over-consumption of oil was demonstrated as positively associated with the risk of type 2 diabetes [44] and GHG emissions in China [45]. Therefore, changing stir-frying and pan-frying into steaming and boiling, which need less oil and are more health-oriented, is of vital importance [46]. The consumption of cereal for diabetes groups complies with the guidance, while the prediabetes groups consumed ca. 65% more than they needed. This finding revealed the neglection of the relationship between cereal and the risk of diabetes for prediabetes people, and enabling those people to receive and highlight popular science knowledge regarding healthy dietary patterns is essential. Previous research has suggested that increasing vegetable and fruit consumption helps with weight loss, which might indirectly reduce type 2 diabetes mellitus incidence [15]. Increasing milk and egg consumption helps with the reduced consumption of meat, which can directly reduce environmental degradation [47,48]. However, our results demonstrated the underconsumption of vegetables, fruit, milk, and eggs. Therefore, a combined scenario that targets increasing the consumption of these food items, and reducing the consumption of meat and cereal, would lead to the multiple benefits of reducing the risk of diabetes and prediabetes and contributing to environmental sustainability [24].

Previous research has demonstrated that a transition from an omnivorous to either a vegan or vegetarian diet would reduce the dietary environmental impacts [30], but it might be contrary to the basic nutritional requirement and human health. However, individual behaviors have an important influence on dietary GHG emissions, which can offset the lower GHG emissions due to the choice of vegetable-origin foods [30]. Therefore, given the huge number of diabetes and prediabetes populations in China, we proposed replacing meat with poultry and reducing the over-consumption of oil and meat, which can result in a trade-off between achieving the healthy goal and protecting the environment. The Chinese Nutrition Society has issued the CDG to popularize nutrition knowledge, guiding people to choose a healthy dietary structure [49,50]. Eating based on the recommended standard of the CDG could reduce undernourishment and stunted development, yet may not significantly reduce the environmental burden. Therefore, another substantial way to reduce the dietary environmental impacts would be through producers, processors, distributors, and retailers. For instance, using more durable packaging and greater coproducts [40]. Furthermore, today’s food supply chain creates ca.13.7 billion metric tons of carbon dioxide equivalents, and the farm stage dominates, representing 61% of food’s GHG emissions [40]. More attention therefore should be paid to the supply side (e.g., producers and supply chain) to address the dilemma of the environment and human health. For instance, increase freshwater aquaculture output, replace meat-based protein with insect-based protein, reduce the use of pesticides, and improve land productivity [51,52]. Policies should also encourage the widespread adoption of certain practices (e.g., conservation agriculture, and organic farming) to reduce the environmental impacts of the farm process [40].

This study primarily investigated the domestic relationships between food consumption, diabetes prevalence, and environmental impacts. Consequently, the EF calculation was based directly on final consumption data rather than on production and trade data. Notably, a significant portion of the soybeans and maize consumed in China are imported from Brazil, primarily due to its superior agricultural yields. [53]. While the yield factor applied in this study is based on global averages, which are lower than Brazil-specific yield data. As a result, the estimated environmental impacts would likely be lower if food imports were explicitly included in the analysis. Building on this insight, future research will aim to examine the extent to which food imports influence both health outcomes and environmental sustainability. Additionally, national composition may influence the results. According to the DE survey, the Hui ethnic group exhibited the lowest diabetes prevalence (6.3%) [22], which may be attributed to their dietary restrictions, specifically the avoidance of pork. In contrast, the lack of significant correlations between diabetes prevalence and the consumption of oil and sugar suggests that culinary preferences may have a limited impact on the findings, given the variation in cooking methods across China—especially regarding the use of seasonings such as oil.

## Conclusion

Food consumption is highly related to human health and environmental degradation [26,54]. The present study presented the triangle relationship across dietary structure, environmental impacts, and the prevalence of diabetes and prediabetes in contemporary China. It concluded that people’s concern for their health helps adjust their dietary structures, which in turn would affect the environment. Consumers’ dietary preferences depend not only on environmental and health issues but also on lifestyles and the related influence factors [30], including disposal income, urban and rural status, geographical locations, and food cultures [3,18,31]. Therefore, understanding the role of lifestyle and food cultures in shaping dietary behavior is crucial for developing targeted strategies that can positively impact both human health and environmental sustainability in the context of sustainable development. Inspired by findings from the health sector [55,56], the development of non-hormonal agents that are both physiologically safe and effective in supporting diabetes management—while simultaneously minimizing environmental impacts—represents a promising avenue for future progress in food science, particularly within interdisciplinary domains bridging medicine and the social sciences. The quantitative examination of the interrelationships among food consumption, human health, and environmental sustainability—utilizing experimental designs with control and comparison groups—constitutes a rigorous and promising interdisciplinary research paradigm that merits systematic exploration within the social sciences.This study remarks on the importance of assessing the health and environmental burden of dietary patterns. It also stresses the necessity of considering approaches directed at reducing diabetes and prediabetes risks and protecting the environment jointly, to take advantage of potential win-win solutions.

## Supporting information

S1 File Dataset. (XLSX)
